# GATA4 Is Required for Budding Morphogenesis of Posterior Foregut Endoderm in a Model of Human Stomach Development

**DOI:** 10.3389/fmed.2020.00044

**Published:** 2020-02-19

**Authors:** Ann DeLaForest, Afiya F. Quryshi, Talia S. Frolkis, Olivia D. Franklin, Michele A. Battle

**Affiliations:** Department of Cell Biology, Neurobiology, and Anatomy, Medical College of Wisconsin, Milwaukee, WI, United States

**Keywords:** gastric development, GATA4, stem cell models, endoderm, morphogenesis

## Abstract

Three-dimensional gastrointestinal organoid culture systems provide innovative and tractable models to investigate fundamental developmental biology questions using human cells. The goal of this study was to explore the role of the zinc-finger containing transcription factor GATA4 in gastric development using an organoid-based model of human stomach development. Given GATA4′s vital role in the developing mouse gastrointestinal tract, we hypothesized that GATA4 plays an essential role in human stomach development. We generated a human induced pluripotent stem cell (hiPSC) line stably expressing an shRNA targeted against GATA4 (G4KD-hiPSCs) and used an established protocol for the directed differentiation of hiPSCs into stomach organoids. This *in vitro* model system, informed by studies in multiple non-human model systems, recapitulates the fundamental processes of stomach development, including foregut endoderm patterning, specification, and subsequent tissue morphogenesis and growth, to produce three-dimensional fundic or antral organoids containing functional gastric epithelial cell types. We confirmed that GATA4 depletion did not disrupt hiPSC differentiation to definitive endoderm (DE). However, when G4KD-hiPSC-derived DE cells were directed to differentiate toward budding SOX2+, HNF1B+ posterior foregut spheroids, we observed a striking decrease in the emergence of cell aggregates, with little to no spheroid formation and budding by GATA4-depleted hiPSCs. In contrast, control hiPSC-derived DE cells, expressing GATA4, formed aggregates and budded into spheroids as expected. These data support an essential role for GATA4 during the earliest stages of human stomach development.

## Introduction

Although gastric diseases cause substantial health and economic burdens worldwide, the fundamental cellular and molecular mechanisms guiding the development and homeostasis of the human stomach remain vastly understudied. One reason likely stems from the diverse anatomical and cellular structures of this organ among various organisms (1, 2). Vertebrate species have evolved stomachs of differing sizes, shapes, and cellular architectures, likely reflecting variations in dietary sources and needs. For example, while the rodent stomach contains a large region of stratified squamous epithelium similar to the esophagus in its proximal domain, the human stomach is completely devoid of such a region. Instead, the human stomach contains only simple columnar epithelium with a unique organization of mature, functionally specific epithelial cells that accomplish the chemical breakdown of food to fuel physiology. The bulk of the human stomach, often referred to as the body, corpus, or fundus (herein referred to as fundus), is similar to the middle region of the rodent stomach. The fundic epithelium in both species is rich in acid-secreting parietal cells, zymogenic chief cells, and protective mucus-secreting cells (1–3). The more distal region of the human and rodent stomach, which leads to the intestine, is termed the antrum. The cellular composition of the antral stomach epithelium varies to a greater extent between rodents and humans than does that of the fundus (3). In rodents, there is a relatively distinct border between fundal and antral regions, with fundic glands being primarily of the oxyntic type (containing digestive parietal and chief cells) and antral glands being primarily enriched with mucus-producing cells and endocrine cells, specifically gastrin producing G-cells. The human antrum, however, is characterized by traditional antral glands, composed of mucus and G-cells, but also contains oxyntic glands, composed of parietal and chief cells, and mixed glands, containing both oxyntic and antral cell types.

**Figure 1:**
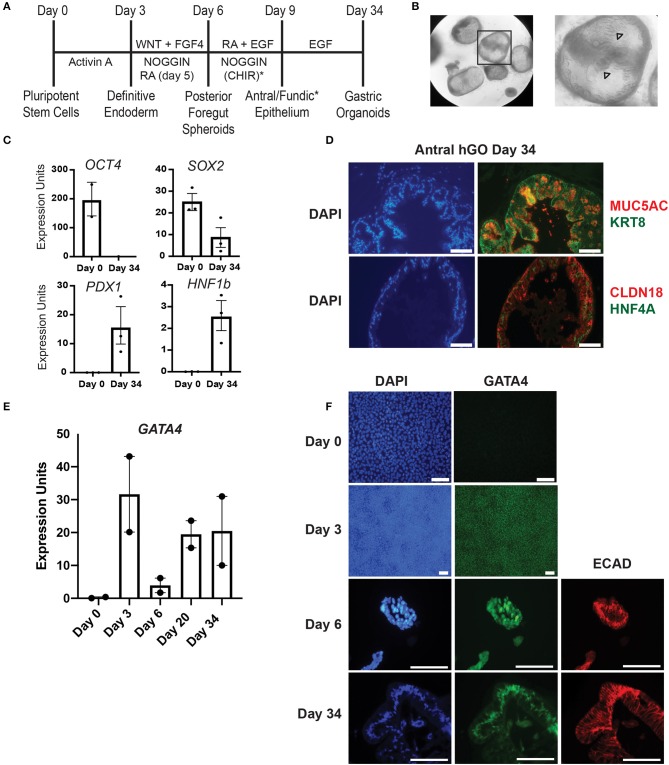
Validation of hiPSC gastric organoid differentiation protocol. **(A)** Timeline delineating the directed differentiation protocol to generate antral or fundic three-dimensional gastric organoids from hiPSCs. The (*) denotes the addition of CHIR to cultures to generate fundic organoids. Additional growth factors, not depicted in this scheme, are required between days 9–34 to generate mature fundic organoids (6, 7). These were omitted because we cultured fundic organoids only to day 10. **(B)** Brightfield images show matrigel embedded gastric antral organoids at day 30 of the human gastric organoid (hGO) differentiation. Arrowheads mark glandular morphology. **(C)** qRT-PCR using RNA from day 0 or day 34 cultures was used to measure the steady-state mRNA expression of transcription factors associated with undifferentiated hiPSCs (*OCT4*) or differentiated human antral gastric organoids (*SOX2, HNF1B*, and *PDX1*). Error bars show SEM. **(D)** Immunofluorescence staining was used to identify proteins characteristic of mature differentiated gastric epithelial cells in day 34 antral hGOs. Left panels show DAPI (blue). Top right panel shows MUC5AC (red) and KRT8 (green); bottom right panel shows CLDN18 (red) and HNF4A (green). Scale bars, 100 μm. **(E)** qRT-PCR was used to measure steady-state mRNA expression of *GATA4* transcript over a time course of antral hGO differentiation. **(F)** Immunofluorescence staining was used to identify GATA4 and E-cadherin proteins over a time course of antral hGO differentiation. Left panels show DAPI (blue); middle panels show GATA4 (green); right panels show E-cadherin (red); Scale bars,100 μm.

Although the structure of the mature gastric organ can differ vastly among species, formative studies in non-human model systems, including the mouse, zebrafish, frog, and chick, have uncovered fundamental pathways that appear to be conserved across species (1, 2, 4, 5). These discoveries informed the development of an *in vitro* human model system, which provides a powerful new vehicle to explore the mechanisms of human gastric development (Figure 1A) (6, 7). Specifically, by treating human pluripotent stem cells first with Activin A and then with WNT, FGF, retinoic acid, and Noggin (to inhibit BMP), the differentiating cell monolayer undergoes morphogenesis, similar to events of early gut tube formation. Three-dimensional free-floating spheroids with posterior foregut endoderm character (SOX2+, HNF1B+) bud from the monolayer, roughly correlating with day E8.5 of mouse development. SOX2+, HNF1B+ posterior foregut endoderm spheroids can be further patterned into fundic or antral type gastric organoids, which contain functional gastric epithelial cell types, by exposing developing spheroids to specific sets of growth factors such as retinoic acid, WNT, EGF, and Noggin. By 13 days of directed differentiation, the developing gastric organoids resemble the mouse E12–14 foregut, containing a pseudostratified gastric-specified epithelium. Between days 13 and 34 of the directed differentiation, which mirrors day E16 through early postnatal development in the mouse, maturation continues such that a simple columnar, glandular-type epithelium containing functional gastric epithelial cell types emerges.

We sought to use this innovative, *in vitro* model system of human gastric development to investigate the role of the key developmental transcription factor GATA4. Previous work from our laboratory demonstrates that GATA4 plays essential roles in mouse small intestinal epithelial patterning and development (8–11). Similar to its expression pattern in the developing and mature mouse small intestine, GATA4 is expressed throughout gastric development and in the mature organ (12–16). GATA4 is present in the posterior foregut endoderm that gives rise to the gastric epithelium. GATA4 expression is maintained in the gastric epithelium throughout embryonic development, and it remains expressed in the mature gastric epithelium. Beyond its expression pattern in the developing and mature stomach, identified more than 25 years ago (15, 16), a functional role for GATA4 in gastric development was demonstrated through a genetic mosaic analysis showing that GATA4 null mouse ES cells failed to contribute to mature differentiated cell types of the mouse stomach of chimeric embryos (12). Therefore, we hypothesized that GATA4 is essential for human gastric development and used the organoid system to test this hypothesis. Studies examining the role of transcription factors and other molecules during the earliest stages of GI development using *in vitro* models such as directed differentiation, as used here, are valuable given that mechanisms of patterning and specification of gut-endoderm derived organs has been challenging to define *in vivo* in model systems due to lack of accessibility (5, 17, 18). For example, GATA4 knockout mice die at gastrulation, well before gastric development (19, 20). Moreover, it can be difficult to identify promoters to drive Cre recombinase with appropriate spatiotemporal dynamics in order to use conditional knockout approaches.

## Methods

### Human Induced Pluripotent Stem Cell (hiPSC) Culture and shRNA Cell Line Generation

Human induced pluripotent stem cell (hiPSC) lines ipsK3 and SV20 [gifts from Stephen Duncan (21, 22)] were maintained on an E-cadherin substrate in mTeSR containing 20% MEF-conditioned media (23–25). SV20 cells were infected with a lentivirus (pLL3.7-Puro) containing a GATA4 shRNA (5′-TGGACATAATCACTGCGTAATTCAAGAGATTACGCAGTGATTATGTCC-3′) at MOI 10, and the population was selected using 1–5 μg/mL puromycin (23). Controls used for differentiation experiments were either uninfected SV20 hiPSCs or SV20 hiPSCs infected with the empty pLL3.7-Puro vector and selected using 1–5 μg/mL puromycin.

### Gastric Organoid Differentiation

hiPSCs were plated at a density of 1.75 × 10^5^ to 2.0 × 10^5^ cells in matrigel-coated 24-well plates with 10 μM Y27632 at day −1. The previously published gastric organoid differentiation protocol was followed (6, 7). Briefly, cells were induced to an endoderm fate using an RPMI-based media containing 100 ng/mL Activin A (R&D Systems) for 3 days, adding 50 ng/mL BMP4 (R&D Systems) only on day 0. Beginning on day 3, endoderm cells were exposed to 500 ng/mL FGF4 (R&D Systems), 2 μM CHIR (Cayman Chemical Company), and 100 ng/mL Noggin (R&D Systems) for 3 days with the addition of 2 μM Retinoic Acid (Sigma) on the last day to induce endoderm cells toward a posterior foregut fate. To induce antral and fundic organoids, day 6 spheroids were embedded in 50 μL matrigel domes (BD Biosciences, 354234) and overlaid with gut media [Advanced DMEM/F12 with N2 (Thermo Fisher), B27 (Thermo Fisher), 2 mM L-glutamine (Thermo Fisher), 10 μM HEPES (Thermo Fisher), penicillin/streptomycin (Thermo Fisher), and 100 ng/ml EGF (R&D Systems)]. For the first 3 days, RA and Noggin were added to the gut media for antral organoids and 2 μM CHIR was added for fundic organoids. Herein, we report data derived from 10 independent differentiations. One differentiation was analyzed only to day 3 with the purpose of validating GATA4 depletion in DE. Nine were analyzed to day 6. Three of those nine were further cultured to day 10 to observe further maturation of spheroids toward a gastric fate. For all 10 differentiations, GATA4 mRNA or protein levels were determined by qRT-PCR, immunoblot, and/or immunofluorescence staining in control and G4KD differentiations at days 3, 4, 5, and/or 6 to validate depletion of GATA4 in experimental cells compared with control cells. Although depletion was validated in all experiments, the method—qRT-PCR, immunoblot, or immunofluorescence staining—varied. The reported budding defect phenotype in GATA4 depleted hiPSCs was consistent among all nine differentiations taken to at least day 6.

### Immunoblot

Nuclear extracts were prepared from differentiated cells using the NE-PER Nuclear and Cytoplasmic Extraction Reagents (Thermo Fisher) and HALT protease inhibitor cocktail (Thermo Fisher). Benzonase (0.5 U/μl) was used to increase protein yield of transcription factors and quality of nuclear extracts. Nuclear proteins (5 μg) were separated using Nu-PAGE Bis-Tris 4–12% gradient gels (Thermo Fisher) and transferred to an Immobilon-FL polyvinylidene difluoride membrane (Millipore). The membrane, exposed to REVERT total protein stain (LI-COR), was imaged, reversed, and washed in 1X PBS before incubating in Odyssey blocking buffer (LI-COR) for 1 h at room temperature. GATA4 antibody (Cell Signaling: 36966, 1:2000, rabbit monoclonal) was added to blocking buffer containing 0.1% Tween and rotated at 4°C overnight. Membranes were washed and exposed to secondary antibody (IRDye 800CW, LI-COR: 926–32213, donkey anti-rabbit, 1:10,000) for 1 h at room temperature. Blots were visualized using an Odyssey Infrared Imaging System (LI-COR) and quantitated using Odyssey software using normalization to REVERT total protein stain.

### RNA Isolation and qRT-PCR

Total RNA was isolated from differentiated cells using the RNeasy mini kit (Qiagen). Complementary DNA (cDNA) was generated from EZ-DNase (Thermo Fisher) treated RNA using MMLV-RT (200 units/μL, Thermo Fisher) with 5 mM dNTPs, 0.1 μg/μL random hexamer primers, and 20 mM DTT or with VILO Master Mix (Thermo Fisher). Transcript levels were measured by TaqMan gene expression assays (Thermo Fisher, *GATA4*: Hs00171403_m1; *SOX17*: Hs00751752_s1; *OCT4*: Hs00999634_gH; *T*: Hs00610080_m1; *PDGFRA*: Hs00998018_m1; *HNF1B*: Hs01001604_m1; *SOX2*: Hs01053049_s1; *PDX1*: Hs00236830_m1; *CDH1* (*E-cadherin)*: Hs01023895_m1; *GAPDH*: 4333764) using TaqMan Gene Expression Master Mix (Thermo Fisher, 4369016) following manufacturer instructions and the CFX384 Touch Real-Time PCR Detection System (Bio-Rad). Data were analyzed as previously described with statistical significance determined using Student's *t*-tests (unpaired, two-tailed) (10).

### Immunofluorescence

Differentiated cells were fixed in 4% paraformaldehyde (Sigma) for 20 min at room temperature. For antigen detection, cells were permeabilized with 0.3% Triton-X for 15 min, blocked with 3% BSA in 1X PBS, and incubated at 4°C overnight with primary antibody in blocking buffer (FOXA2, Novus:H00003170-M12, 1:250, mouse monoclonal; GATA4, Cell Signaling: 36966, 1:500, rabbit monoclonal; E-Cadherin, BD Bioscience: 610181,1:4,000, mouse monoclonal; KRT8, DSHB, TROMA-I,1:250, rat monoclonal; CLDN18, Sigma: HPAO 18446, 1:200, rabbit polyclonal; MUC5AC, Thermo Fisher: MA1-38223, 1:100, mouse monoclonal; HNF4A, Santa Cruz: SC-6556, 1:250, goat polyclonal). Cells were washed three times with 1X PBS and incubated with DAPI and appropriate Alexa-Fluor secondary antibodies (Alexa-Fluor 488 Donkey anti-goat, Thermo Fisher: A11055,1:500; Alexa-Fluor 488 Donkey anti-rat, Thermo Fisher: A21208,1:500; Alexa-Fluor 488 Donkey anti-rabbit, Thermo Fisher: A21206,1:500; Alexa-Fluor 594 Donkey anti-rabbit, Thermo Fisher: A21207,1:500; Alexa-Fluor 594 donkey anti-mouse, Thermo Fisher: A21203,1:500) Micrographs were captured using an Eclipse TE300 fluorescent microscope (Nikon) and Spot Camera software. Images were assembled into figures using Adobe Photoshop and Illustrator, and images from control and experimental samples were processed identically. To measure E-Cadherin fluorescence intensity, three random fields per sample were captured and red immunofluorescence pixel intensity was quantified using ImageJ software. Data were compiled from three independent differentiations.

## Results

### Human Induced Pluripotent Stem Cell (hiPSC) Derived Gastric Organoids Express GATA4 and Other Mature Gastric Epithelial Cell Markers

The directed differentiation of hiPSCs into three-dimensional gastric organoids containing a functional gastric epithelium and supporting mesenchymal cells has been shown to parallel the *in vivo* process of gastric development observed in mice and other non-human model organisms (2, 6, 7). We used this innovative directed differentiation paradigm to explore the role of the zinc finger containing transcription factor GATA4 in human gastric development. Using wild-type hiPSCs, we first validated key milestones throughout the differentiation protocol established by McCracken and colleagues (6, 7) (Figure 1A). hiPSC-derived definitive endoderm (DE), generated by treatment of hiPSCs with Activin A, was differentiated to a posterior foregut fate by activating WNT and FGF signaling and by inhibiting BMP signaling via Noggin. Budding, free-floating three-dimensional posterior foregut spheroids were expanded and matured by embedding in matrigel and treating with appropriate antral differentiation growth factor cocktails. After 30 days of directed differentiation toward an antral fate, the resulting gastric antral organoids displayed a complex folded and glandular architecture as described previously [Figure 1B, (6)]. Expression of *OCT4*, a pluripotency marker abundant in the starting hiPSC population, was absent at day 34, as expected (Figure 1C). Conversely, at day 34, organoids expressed transcription factors expected to be present in the mature gastric epithelium, including *SOX2, HNF1B*, and *PDX1* (Figure 1C). The presence of *PDX1* confirms antral identity (3, 6). Furthermore, after 34 days in culture, gastric organoids expressed proteins present in differentiated gastric epithelial cells *in vivo*, including MUC5AC, KRT8, CLDN18, and HNF4A (Figure 1D). These data demonstrate that we successfully replicated the gastric directed differentiation protocol first described by McCracken and colleagues (6, 7), taking hiPSCs from pluripotency to three-dimensional gastric organoids with mature gastric epithelial cell types.

Given that our goal was to examine the role of GATA4 in the process of human gastric development, we examined the status of GATA4 expression over the differentiation time course looking at steady-state mRNA and protein levels via qRT-PCR and immunostaining. We observed that GATA4 transcript was expressed in definitive endoderm (day 3), as expected, and that it remained present throughout the entirety of the differentiation protocol (Figure 1E). While undifferentiated hiPSCs lack GATA4 protein (Figure 1F, day 0), nearly all definitive endoderm cells expressed GATA4 protein (Figure 1F, day 3). GATA4 protein was also identified within posterior foregut spheroids (Figure 1F, day 6), demonstrating that although there is a drop in the abundance of *GATA4* mRNA in the total population of differentiating cells at day 6 (Figure 1E), GATA4 protein remains expressed in the posterior foregut spheroid endoderm. By day 34, the gastric organoids contained GATA4 positive cells lining the organoid lumen. Co-expression of E-cadherin at days 6 and 34 confirmed that GATA4 expressing cells were epithelial (Figure 1F). These results demonstrate that similar to the course of mouse gastric development, GATA4 remained present throughout all stages of human gastric differentiation, being detected in definitive endoderm, posterior foregut endoderm, and mature gastric organoids. Therefore, GATA4 is a crucial molecule to investigate in human gastric development.

### GATA4-Depleted Human Induced Pluripotent Stem Cells (hiPSCs) Generated Definitive Endoderm Efficiently

**Figure 2:**
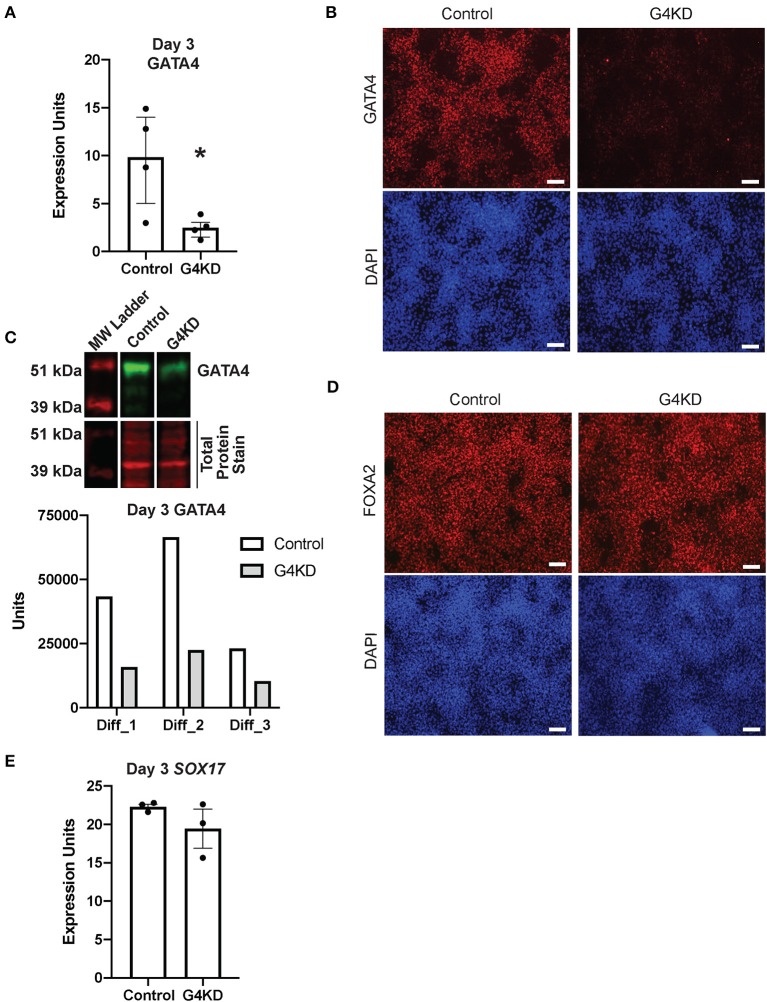
GATA4-depleted hiPSCs efficiently differentiated into DE. **(A)** qRT-PCR was used to measure steady-state *GATA4* transcript at day 3 of endoderm differentiation for pLL3.7-Puro control hiPSCs and G4KD hiPSCs. *n* = 4 independent differentiations. Error bars show SEM. Statistical significance was determined by Student's *t*-test. *p < 0.05. **(B)** Immunofluorescence staining was used to measure GATA4 protein (red) at day 3 of endoderm differentiation for pLL3.7-Puro control hiPSCs and G4KD hiPSCs. DAPI (blue) shows nuclei. Image shown is representative of four independent differentiations. Scale bars, 100 μm **(C)** Immunoblotting was used to quantify GATA4 protein (green) from nuclear extracts of pLL3.7-Puro control hiPSCs and G4KD hiPSCs isolated at day 3 of endoderm differentiation. The blot shown is representative of three independent differentiations. Quantification of GATA4 protein in each of the three independent differentiation experiments is graphed. **(D)** Immunofluorescence staining was used to identify FOXA2 protein (red) in pLL3.7-Puro control hiPSCs and G4KD hiPSCs at day 3 of the endoderm differentiation. DAPI (blue) shows nuclei. Scale bars, 100 μm. **(E)** qRT-PCR was used to measure steady-state *SOX17* transcript at day 3 of endoderm differentiation in pLL3.7-Puro control hiPSCs and G4KD hiPSCs, *n* = 3 independent differentiations. Error bars represent SEM.

To test the extent to which GATA4 is required for human stomach development *in vitro*, we established a polyclonal GATA4-depleted hiPSC line by infecting undifferentiated SV20 hiPSCs with a lentiviral vector containing an shRNA targeted against GATA4 and a puromycin resistance gene (pLL3.7-Puro-G4KD, herein denoted as G4KD-hiPSCs) (23). We differentiated G4KD-hiPSCs and control hiPSCs, either SV20 hiPSCs or a polyclonal SV20 hiPSC line generated by infecting undifferentiated cells with a lentiviral vector containing the puromycin resistance gene but lacking the GATA4 shRNA (pLL3.7-Puro), to gastric spheroids. For this study, control hiPSCs and G4KD-hiPSCs were differentiated in 10 independent experiments, and GATA4 depletion was measured in each experiment by qRT-PCR, immunoblot, and/or immunofluorescence staining. *GATA4* transcript was depleted by nearly 70% (range 60–85%) in G4KD-hiPSC-derived endoderm compared with control hiPSC-derived endoderm (Figure 2A). Immunofluorescence staining for GATA4 protein showed that GATA4 was depleted in the majority of cells within the polyclonal G4KD-hiPSC population (Figure 2B). Using immunoblot, we estimated that GATA4 protein was depleted by an average of 61% (range 55–66%) in G4KD-hiPSC endoderm compared with control endoderm (Figure 2C). Although the maximal level of GATA4 protein or mRNA in control hiPSC differentiated cultures varied from experiment to experiment, a consequence of the inherent variability in differentiation efficiency from experiment to experiment, GATA4 mRNA and protein were both nevertheless consistently decreased (Figures 2A–C). In sum, we evaluated GATA4 depletion in 10 independent differentiations using a variety of methods and found in each experiment that GATA4 was depleted in G4KD-hIPSCs compared with matched control cells.

GATA4 is dispensable during DE differentiation (23). Therefore, to validate that the G4KD-hiPSC line generated in our laboratory was similarly capable of generating DE, we examined two transcription factors essential for DE differentiation, FOXA2 and SOX17 (6, 7, 26), at day 3. The majority of cells were positive for FOXA2 in both the control and G4KD-hiPSC DE populations (Figure 2D). Additionally, *SOX17* transcript was expressed consistently between G4KD-hiPSC and control DE populations (Figure 2E). From these data, we conclude that G4KD-hiPSCs, depleted in GATA4, maintained the ability to efficiently differentiate to DE, allowing for examination of the role of GATA4 in subsequent steps in gastric differentiation.

### Budding Morphogenesis Was Disrupted in GATA4-Depleted Human Induced Pluripotent Stem Cells (hiPSCs)

**Figure 3:**
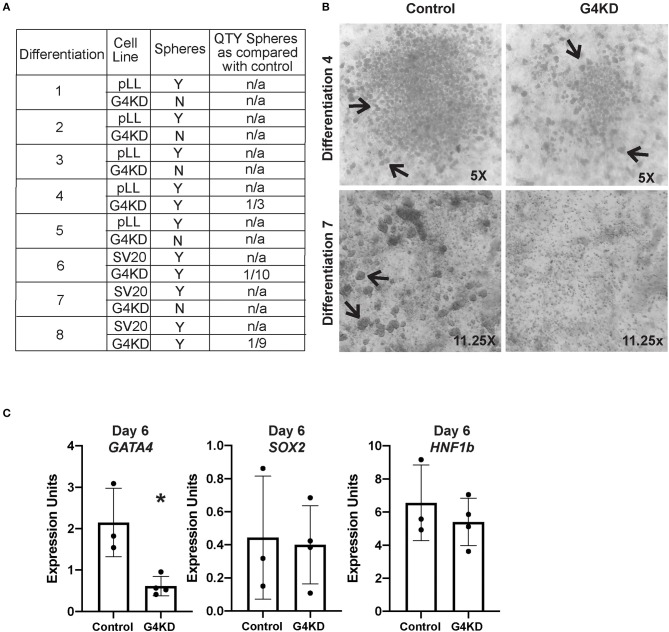
GATA4-depleted hiPSCs failed to form posterior foregut spheroids in culture. **(A)** Table reports the outcome of eight independent gastric differentiations with two control (SV20 and pLL3.7-Puro) hiPSC lines and the G4KD-hiPSC line. Spheroids were present in all control differentiations. The G4KD-hiPSC produced reduced spheroid numbers at day 6 of the gastric organoid directed differentiation. In five differentiations, no floating budded spheroids were observed. In three differentiations, fewer floating budded spheroids were observed. **(B)** Upper panel shows a representative differentiation (#4) in which a reduced number of spheroids was observed. Lower panel shows a representative differentiation (#7) in which no floating budded spheroids were observed. Black arrows indicate spheroids in both panels. **(C)** qRT-PCR was used to measure steady-state mRNA levels of *GATA4, HNF1B*, and *SOX2* transcripts in spheroids isolated from day 6 of differentiation, *n* = 3 independent differentiations for control hiPSCs and 4 independent differentiations for G4KD-hiPSCs. Error bars represent SEM. Statistical significance was determined by Student's *t*-test. *p < 0.05.

During hiPSC differentiation to gastric organoids, GATA4+ endodermal cells condense into three-dimensional mounds and bud into the differentiation media, paralleling *in vivo* gut tube morphogenesis (5–7). Directed differentiation of control hiPSCs generated free-floating buds at day 6 of the differentiation, as expected (Figures 1F, 3A,B). GATA4-depleted endodermal cells, however, formed fewer spheroids compared with control cells. In five of eight gastric differentiations, G4KD-hiPSC cultures generated no floating spheroids (Figures 3A,B). In the remaining three gastric differentiations, G4KD-hiPSCs generated at most one-third the number of spheroids formed by control cells (Figures 3A,B). These data provide evidence that GATA4 is required for budding morphogenesis of posterior foregut spheroids.

To determine if spheroids generated from the G4KD-hiPSCs were correctly specified to posterior foregut, we examined expression of *HNF1B* and *SOX2*, two transcription factors known to demarcate this developmental milestone (5–7). We observed no difference in expression of the transcript of either factor when GATA4 was depleted, suggesting that spheroids emerging from GATA4-depleted endodermal cells were indeed posterior foregut (Figure 3C). We further validated that spheroids emerging from G4KD-hiPSC-derived endoderm maintained depleted GATA4 levels by examining GATA4 transcript abundance in control and G4KD-hiPSC spheroids at day 6. We observed that G4KD-hiPSC derived posterior foregut spheroids retained diminished levels of GATA4 transcript compared with control spheroids (Figure 3C).

To examine the propensity of spheroids formed by GATA4-depleted endodermal cells to mature into antral or fundic gastric organoids, we embedded day six spheroids into three-dimensional matrigel domes and applied appropriate antral or fundic growth factor cocktails (Figure 1A). After 4 days, control spheroids directed into either antral or fundic fate displayed smooth outer edges and remained spherical (Supplemental Figure 1). In contrast, GATA4-depleted spheroids lost smooth outer edges and appeared to disintegrate within the matrigel domes, suggesting that spheroids generated from GATA4-depleted endodermal cells were not competent to mature as antral or fundic gastric organoids (Supplemental Figure 1).

**Figure 4:**
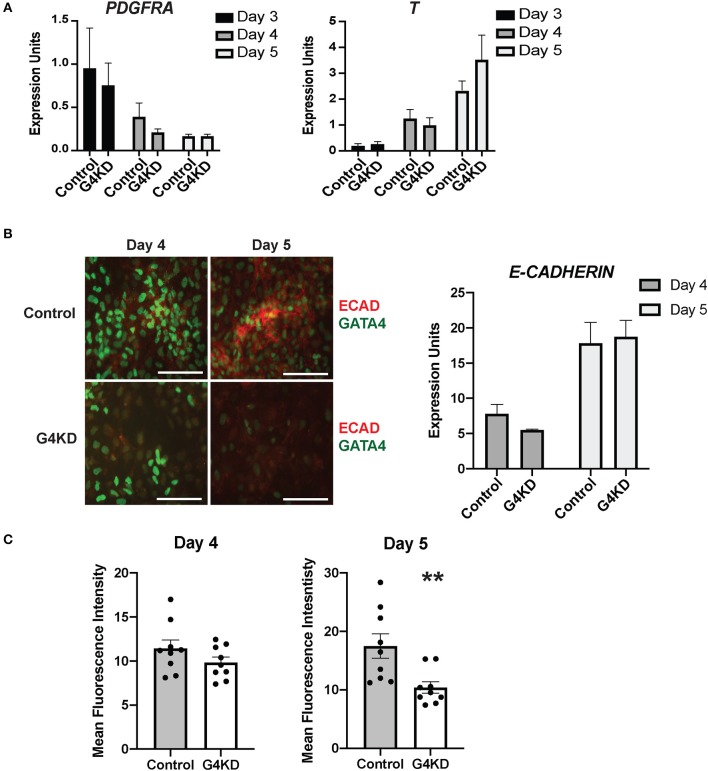
E-cadherin protein was reduced in differentiating GATA4-depleted hiPSCs during posterior foregut specification and patterning compared with control hiPSCs. **(A)** qRT-PCR was used to measure the steady-state mRNA levels of the mesenchymal marker *PDGFRA* and the mesoderm marker *T* (brachyury). *n* = 3 independent differentiations with pLL3.7-Puro control hiPSC and G4KD hiPSC lines. Error bars show SEM. **(B)** Immunofluorescence staining was used to measure E-cadherin (red) and GATA4 (green) proteins at days 4 and 5 of posterior foregut differentiation of pLL3.7-Puro control hiPSC and G4KD hiPSC lines. Scale bar, 100 μm. Images are representative of three independent differentiations with each line. qRT-PCR was used to measure steady-state mRNA levels of E-cadherin in control hiPSC and G4KD-hiPSCs at days 4 and 5. *n* = 3 independent differentiations with pLL3.7-Puro control hiPSC and G4KD hiPSC lines. Error bars show SEM. **(C)** Quantification of red immunofluorescence pixel intensity for E-cadherin protein at days 4 and 5 of posterior foregut differentiation of pLL3.7-Puro control hiPSCs and G4KD hiPSCs. ImageJ was used to quantify intensity from three random fields for each sample in three independent differentiations. Error bars show SEM. Statistical significance was determined by Student's *t*-test. **p < 0.01.

Budding morphogenesis has been shown to occur in several other gut tube organoid differentiation protocols, and these studies have shown that the mesenchymal population within the differentiation culture is required (5, 26, 27). *In vivo* studies have also shown that gastric specification requires mesenchyme (1, 2, 4, 28). Therefore, to examine the idea that loss of GATA4 alters the mesenchymal cell population, we measured transcript levels for two genes encoding proteins with essential roles in mesendoderm to mesoderm and mesenchyme transition, *T* and *PDGFRA* (29, 30). We found no change in levels of these transcripts across days 3–5 of the differentiation (Figure 4A), suggesting that the mesenchymal population was similarly present in both control and GATA4-depleted differentiations.

### Loss of GATA4 During Posterior Foregut Specification Decreases the Quantity of E-Cadherin in Cultures

Finally, we examined the level of E-cadherin protein in differentiating cultures during the stage of posterior foregut endoderm specification and initiation of budding morphogenesis. We performed immunofluorescence co-staining for GATA4 and E-cadherin proteins at days 4 and 5 in three independent differentiations. Overall, levels of E-cadherin protein appeared diminished in GATA4-depleted cultures at both time points with a more striking difference observed at day 5 (Figures 4B,C). E-cadherin was depleted in G4KD-hiPSC cultures, even in regions that maintained GATA4+ cells. To quantify E-cadherin protein abundance in G4KD-hiPSC and control differentiated cultures, we used ImageJ software to measure fluorescence intensity. We measured pixel intensity in three random fields from each day over three independent differentiation experiments. We observed that E-cadherin signal was less abundant in G4KD-hiPSC differentiations at day 5 (Figure 4C). We examined the steady-state *E-cadherin* mRNA level in cells at days 4 and 5. We found no corresponding change in mRNA levels between groups (Figure 4B). These data suggest that the epithelial cell differentiation program was abnormal upon loss of GATA4. Moreover, with no detected change in *E-cadherin* mRNA, the data suggest that E-cadherin was affected at the protein level (i.e., translation, stability, trafficking, and/or degradation). Although G4KD-hiPSCs appear to be specified to posterior foregut fate, as determined by HNF1B and SOX2 expression, the change in E-cadherin protein could represent a mechanistic aspect of the general morphogenetic failure of the GATA4-depeleted cells to bud at day six.

## Discussion

Here, using an *in vitro* model of human gastric development, we identify an essential role for GATA4 during the earliest stages of gastric morphogenesis. Approaches in other model systems, including rodents, chicks, and fish, have not been sufficient to query the function of GATA4 at this early stage of development because of technical limitations in eliminating GATA4 protein during this early period of gut tube development. These studies highlight the power of *in vitro* differentiation systems to examine processes that have been difficult to approach in other model systems. Because human pluripotent stem cells can be differentiated, processes of human development, which are otherwise inaccessible, can be modeled and studied.

Although our work highlights a provocative phenotype, the mechanisms underlying how GATA4 directly influences spheroid generation and maturation remain to be determined. Spheroids may fail to form because GATA4 plays a vital role in integrating the signaling pathways received by endodermal cells to program maturation beyond initial tissue-fate specification. Therefore, when GATA4 is depleted, the signals received by the nascent posterior foregut endodermal cells fail to translate into spheroid morphogenesis. Precedence for GATA4 crosstalk with WNT and FGF signaling pathways has been suggested in studies in intestinal and cardiac development and cardiac reprogramming protocols (8, 31–34).

Beyond the endodermal cells, budding morphogenesis in other *in vitro* systems that model human GI development has been shown to require the mesenchymal population within the differentiation culture (5, 26, 27). *In vivo* studies also revealed that gastric development requires mesenchyme (1, 2, 4, 28). Our data suggest that mesenchymal cell numbers are similar between control and GATA4-depleted differentiations. Our data, however, cannot rule out the possibility that the mesenchymal cells in GATA4-depleted cultures are functionally defective. Abnormal endoderm cells with decreased levels of GATA4 may fail to secrete sufficient levels of crucial signaling factors, thereby disrupting endoderm-mesoderm crosstalk and morphogenesis.

Most compelling among our data to explain the budding defect stems from our finding that E-cadherin protein abundance is lower within differentiating GATA4-depleted cultures compared with control cultures during the period in which morphogenetic processes are initiating. Lower levels of E-cadherin in endodermal cells would likely compromise endodermal cell-to-cell adhesion. A loss of cohesion among the endodermal cells could translate into morphogenetic defects, including defective budding. Moreover, studies in *Drosophila* focused on understanding E-cadherin protein function during cellular migration events of fly midgut development uncovered a paradigm-shifting function of E-cadherin in mesenchymal cells that could be at play in this system (35). In this study, mesenchymal cells in the posterior midgut of E-cadherin mutant flies detach from each other and neighboring endodermal cells. The consequence is lost coordination between the endodermal and mesodermal cell populations causing disordered cohesion and migration during midgut morphogenesis. Loss of E-cadherin in developing mesenchymal cells in GATA4-depleted cultures may contribute to the failure of the cultures to bud posterior endoderm gut spheroids. Although E-cadherin is typically associated with endodermal and endoderm-derived epithelial cell populations, mesendoderm cells, the precursors to endodermal and mesodermal cells express E-cadherin (30).

It was also intriguing that we noted a substantial decrease in GATA4 transcript levels as cultures transitioned from definitive endoderm to posterior foregut endoderm (day 3–day 6). Later in the differentiation process, GATA4 transcript levels rose (day 6–days 20 and 34). The presence of Noggin in the differentiation media could explain the downregulation of *GATA4* transcript between days 3 and 6. In cardiac development, Noggin represses GATA4 transcription (33). The facts that Noggin is required to specify posterior foregut endoderm and that Noggin can repress GATA4 suggest that a precise level of GATA4 is required for posterior foregut development. G4KD-hiPSCs that succeeded in budding likely expressed GATA4 levels at or above a required threshold set by Noggin. Later, however, G4KD-hiPSCs buds failed to mature, suggesting that the level of GATA4 in these cells, although sufficient to allow budding, was insufficient to allow for subsequent maturation. Studies in the mouse align with the failure of GATA4-depleted spheroids to mature as GATA4 was shown to regulate gastric epithelial cytodifferentiation (12).

In summary, this work describes an exciting new phenotype related to GATA4′s role in the earliest stages of gastric development using a model of human gastric development. These studies advance our knowledge of GATA4 function during gastrointestinal development. To date, the role of GATA4 in the early stages of GI development, including gastric development, has not been examined, mainly due to technical limitations in approaches with commonly used model systems. *Gata4* null mice die before gastric development, and conditional knockout approaches at the earliest stages of endoderm specification have not been explored. Future studies will examine, in greater depth, the mechanistic roots of the phenotype. Moreover, experiments with hiPSC lines capable of expressing exogenous GATA4 at discrete time points will allow us to move past the early developmental defects and more fully examine the role of GATA4 in human gastric epithelial cell maturation and function. These studies will be significant given that GATA4 has been implicated in gastric diseases, including gastric cancer. Finally, delineating the role of factors such as GATA4 during the specification of endoderm into the gastric fate will assist in cell-based regenerative therapies and possibly biomarker identification.

## Data Availability Statement

All datasets generated for this study are included in the article/Supplementary Material.

## Author Contributions

AD and MB conceived the study, interpreted the data, and wrote the manuscript. AD, AQ, TF, and OF performed experiments. AD analyzed all data. MB provided project oversight and provided financial support for the studies. All authors read and approved the final manuscript.

### Conflict of Interest

The authors declare that the research was conducted in the absence of any commercial or financial relationships that could be construed as a potential conflict of interest.

## Supplementary Material

Supplemental Figure 1 Representative micrographs of matrigel embedded posterior foregut spheroids from control pLL3.7-Puro SV20 hiPSCs and G4KD hiPSCs at day 10 of the differentiation. **(A)** Representative micrographs of embedded antral and fundic spheroids formed by pLL3.7-Puro control hiPSCs and G4KD hiPSCs at day 10 of the differentiation protocol. **(B)** Higher magnification images of micrographs shown in **(A)** to illustrate rounded mature spheroids in differentiations of pLL3.7-Puro control hiPSCs and the disintegrating spheroids in G4KD hiPSCs differentiations.
