# Supplementary figures and images for: GATA4 Is Required for Budding Morphogenesis of Posterior Foregut Endoderm in a Model of Human Stomach Development
Source: Front Med (Lausanne). 2020 Feb 19;7:44. doi: 10.3389/fmed.2020.00044 (PMC7042400; doi:10.3389/fmed.2020.00044)

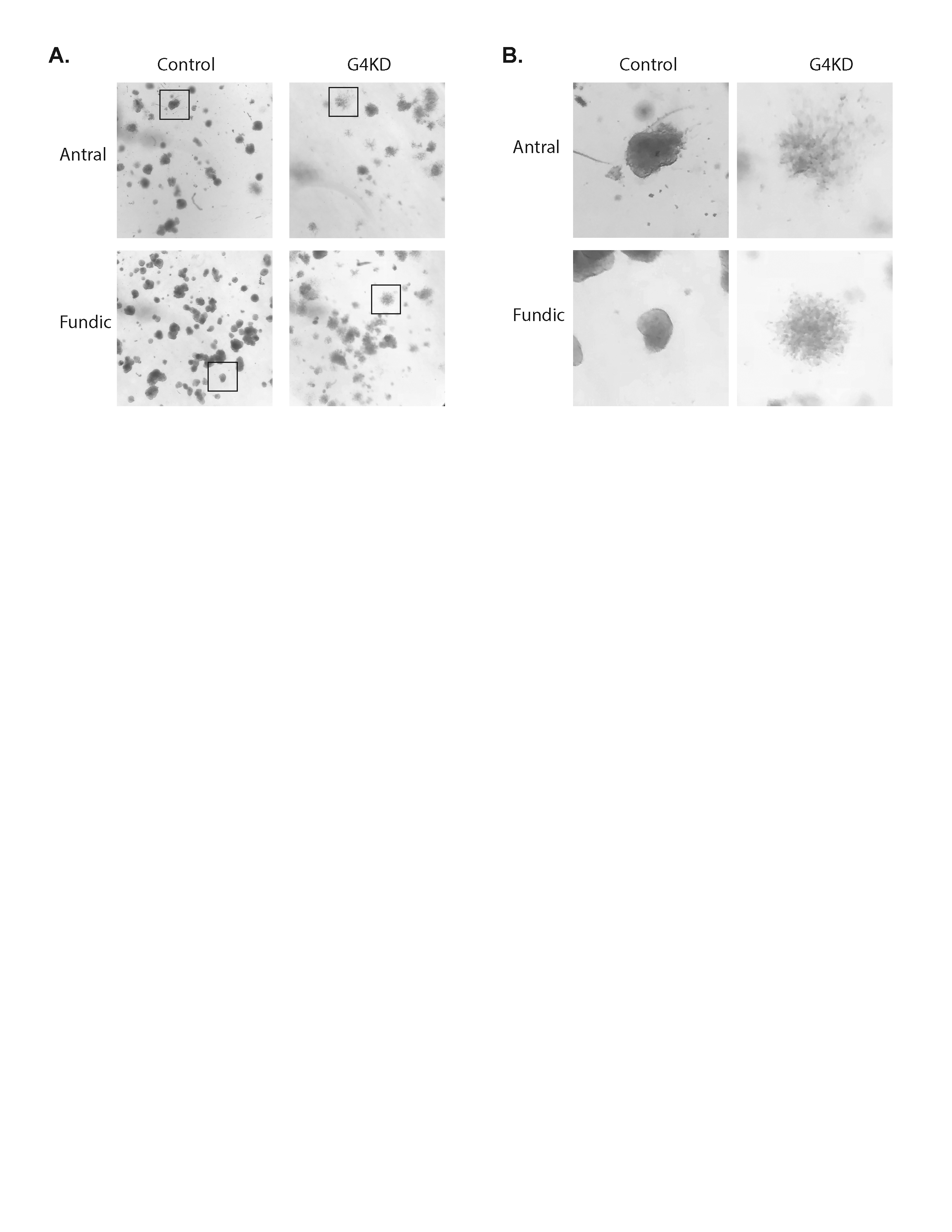

Supplement: Supplemental Figure 1 — Representative micrographs of matrigel embedded posterior foregut spheroids from control pLL3.7-Puro SV20 hiPSCs and G4KD hiPSCs at day 10 of the differentiation. (A) Representative micrographs of embedded antral and fundic spheroids formed by pLL3.7-Puro control hiPSCs and G4KD hiPSCs at day 10 of the differentiation protocol. (B) Higher magnification images of micrographs shown in (A) to illustrate rounded mature spheroids in differentiations of pLL3.7-Puro control hiPSCs and the disintegrating spheroids in G4KD hiPSCs differentiations. [file Image_1.TIF]
